# Potential New H1N1 Neuraminidase Inhibitors from Ferulic Acid and Vanillin: Molecular Modelling, Synthesis and in Vitro Assay

**DOI:** 10.1038/srep38692

**Published:** 2016-12-20

**Authors:** Maywan Hariono, Nurshariza Abdullah, K.V. Damodaran, Ezatul E. Kamarulzaman, Nornisah Mohamed, Sharifah Syed Hassan, Shaharum Shamsuddin, Habibah A. Wahab

**Affiliations:** 1 School of Pharmaceutical Sciences, Universiti Sains Malaysia, 11800 Minden, Pulau Pinang, Malaysia; 2 School of Health Sciences, Universiti Sains Malaysia, 16150 Kubang Krian, Kelantan, Malaysia; 3 Jeffry Cheah School of Medicine and Health Sciences, Monash University Malaysia, 47500 Bandar Sunway, Selangor Darul Islam, Malaysia; 4 Malaysian Institute of Pharmaceuticals and Nutraceuticals, Ministry of Science, Technology and Innovation, Halaman Bukit Gambir, 11900 Bayan Lepas, Pulau Pinang, Malaysia

## Abstract

We report the computational and experimental efforts in the design and synthesis of novel neuraminidase (NA) inhibitors from ferulic acid and vanillin. Two proposed ferulic acid analogues, **MY7** and **MY8** were predicted to inhibit H1N1 NA using molecular docking. From these two analogues, we designed, synthesised and evaluated the biological activities of a series of ferulic acid and vanillin derivatives. The enzymatic H1N1 NA inhibition assay showed **MY21** (a vanillin derivative) has the lowest IC_50_ of 50 μM. In contrast, the virus inhibition assay showed **MY15**, a ferulic acid derivative has the best activity with the EC_50_ of ~0.95 μM. Modelling studies further suggest that these predicted activities might be due to the interactions with conserved and essential residues of NA with ΔG_bind_ values comparable to those of oseltamivir and zanamivir, the two commercial NA inhibitors.

In the last decade, the world was threatened with the emergence of pandemic influenza virus. A highly pathogenic influenza (H5N1) transmission from birds to human that resulted in 43 deaths in Vietnam, Indonesia, China, Cambodia and Thailand shocked the world in 2005^1^. More deaths were reported in the subsequent years and the threat of H5N1 was further compounded by the emergence of H1N1 pandemic in 2009^2^. The World Health Organization (WHO) confirmed that the pandemic spread to over 220 countries with more than 39 million cases and 15,417 deaths worldwide as reviewed^3^.

There are vaccines to prevent the influenza infection and antiviral drugs for the treatment are also available. However, the existing vaccines have been mostly ineffective due to the emergence of mutations^4^. The use of M2 channel blockers such as amantadine and rimantadine has been limited due to drug resistance problems and side effects. Thus, the current frontline drugs for influenza infection have been limited to neuraminidase inhibitors such as oseltamivir (**OTV**) and zanamivir (**ZNR**).

Neuraminidase (NA), a surface glycoprotein vital for the viral replication is an important target for anti-influenza drug^5^. Although **ZNR** is highly effective, its inhalational delivery^6^,^7^ is not very attractive as oral delivery (via capsule/tablet) is generally more preferable. **OTV** overcomes this limitation, but the production cost is quite high as it relies on the expensive starting material, shikimic acid^8^. Furthermore, the currently circulating clinical H274Y H1N1 mutant is quite resistant to **OTV**^9^,^10^ and this might be one of the reasons for the fast track approval for laninamivir^11^.

Many efforts have been made to discover new NA inhibitors with various scaffolds, including aromatic^12^,^13^, dihydropyrane^14^,^15^, cyclopentane^16^, cyclohexene^17^,^18^, pyrrolidine^19^ and others^20^. There are also many natural product compounds reported to have anti-NA activity^21^. In our recent virtual screening study, we identified *Garcinia mangostana* among the five Malaysian plants that have anti-H5N1 NA activity^22^. In the initial phase of our study, we managed to isolate ferulic acid (**FA**) from *G. mangostana* which demonstrated a sensible inhibition toward H1N1 NA with an IC_50_ of 140 μM. However, in the subsequent extraction, we failed to reisolate the compound. Since **FA** was not ranked in our top 100 virtual hits, and it is commercially available, we did not pursue with the isolation. Instead, we decided to perform a thorough molecular modelling to understand better its binding to the NA in our quest to design and synthesize potential analogues as NA inhibitors. The structure of **FA** comprises three functional groups which could probably contribute to the interaction with H1N1 NA, i.e. the carboxylate, hydroxy, and methoxy groups. Furthermore, the ring system of this aromatic compound is more planar than that of shikimic acid of **OTV**. Conformationally flexible compounds in a free state lose energy upon binding to the macromolecule. Introduction of a planar aromatic structure will reduce the flexibility of a compound and will not lose as much entropy upon binding. This favourable entropy generally increases ligand-receptor binding affinity. Furthermore, the prevalence of aromatic in drug molecules has been attributed to a feasible synthesis. Making compounds with aryl-aryl systems are more time and cost effective as reviewed^23^. Thus, we found that **FA** to be an interesting scaffold for further designs of novel NA inhibitors.

Ferulic acid has a highly correlated structure with vanillin, **VN**. It can be prepared synthetically by reacting **VN** and malonic acid. *Vice versa*, **VN** can be produced by hydrolysing **FA** at certain condition^24^,^25^,^26^. In this present study, the goal is to synthesise analogues from **FA** and **VN** to produce potential NA inhibitors aided by molecular docking. The inhibition of H1N1 NA was validated using the *in vitro* enzymatic and viral inhibition studies. It is hoped that the results from this study would provide an insight into the design of novel and more potent NA inhibitors.

## Results and Discussion

### Molecular Modelling

**Figure 1:**
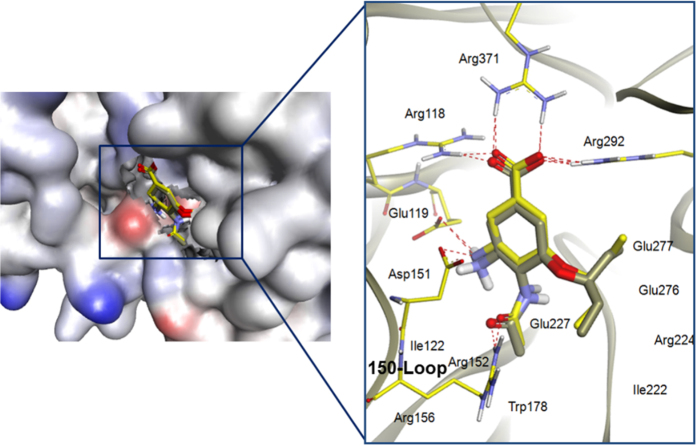
Superimposition of the docked and crystallographic oseltamivir poses (grey and yellow carbons, respectively) showing that the interacting residues are identical for both poses.

**Figure 2:**
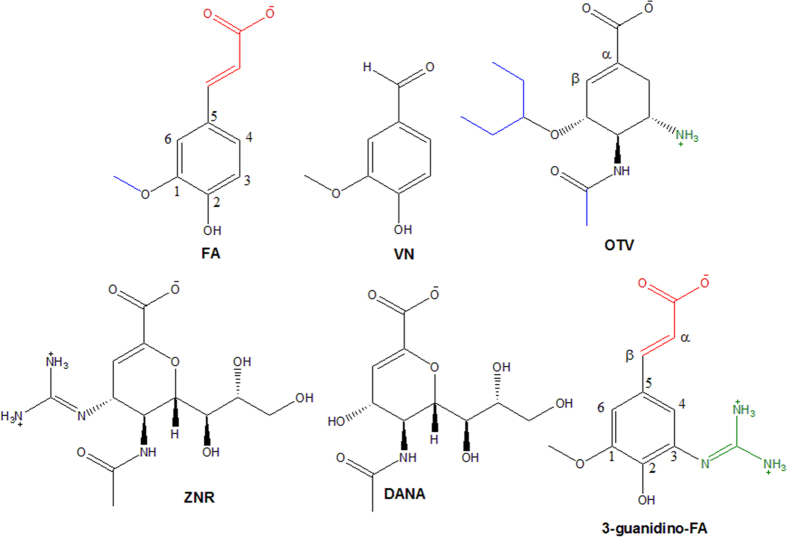
Proposed NA inhibitors (**FA**, **VN** and **3-guanidino-FA**) and NA Inhibitors (**OTV, ZNR** and **DANA**), *β*–unsaturated bond, hydrophobic and basic functional groups are colored by red, blue and green, respectively. The structures were drawn using ChemDraw Ultra 8.0 (www.chembridgesoft.com).

The docking protocol was validated by redocking oseltamivir, **OTV** to its co-complex 2009 H1N1 NA crystal structure (PDB ID: 3TI6)^27^. The result showed that the redocked OTV pose was similar to the crystallographic pose with an RMSD of 0.515 Å (see Fig. 1) indicating that the AutoDock docking parameters used are applicable to this system. Figure 2 showed the structures of the currently well-known neuraminidase inhibitors Oseltamivir (OTV), Zanamivir (ZMR) and 2-deoxy-2,3-didehydro-N-acetylneuraminic acid or Neu5Ac2en (DANA) as well as Ferulic acid (FA) and Vanillin (VN). For ease of comparison, the six carbon atoms in the benzene ring are numbered in correspondence to the carbon atoms of the alicyclic ring (of the shikimic acid scaffold) of OTV.

FA has a ring structure similar to OTV, despite its ring structure is planar and aromatic as opposed to non-planar cyclohexene ring of OTV. Both FA and OTV have alkoxy group (methoxy in FA and isopentoxy in OTV) at C1. This feature is suggested to interact with the nonpolar side chains of Ile222, Arg224 and Glu276 in the hydrophobic pocket of NA^28^. At C5, there is also a similar feature between FA and OTV in term of the presence of carboxylate group (FA–propenoate and in OTV–methanoate) which may contribute to either electrostatic or hydrogen bond interactions (HBA/HBD). The hydrophobic character of acetamido in **OTV** at C2 position is necessary to interact with the Trp178’s indole side chain. In contrast, at this C2 position, **FA** has a hydrophilic character (OH group). The basic character of the amino group in **OTV** at position C3 is suggested to fit into the acidic pocket near the 150-loop, which also plays an essential role in NA’s activity^29^. However, there is no basic character at the corresponding position in **FA**. Therefore, slight structural modifications of ferulic acid are predicted to improve the **FA** activity.

Introducing an amino substituent of **FA** with a guanidino group is believed to improve the *in vitro* NA inhibition as exemplified by the rationale for designing **ZNR** from **DANA (**Neu5Ac2en), see Fig. 2. **ZNR** has K_i_ of 250 magnitudes lower than its analogue 4-amino-**DANA**^30^. It was further shown that **ZNR** with its guanidino group was able to lock the acidic pocket by interacting with Asp151 near 150–cavity more intensively than **OTV**^31^. This shows how important the guanidino group in NA inhibition which prompted us to introduce a guanidino group at the position C3.

**Figure 3:**
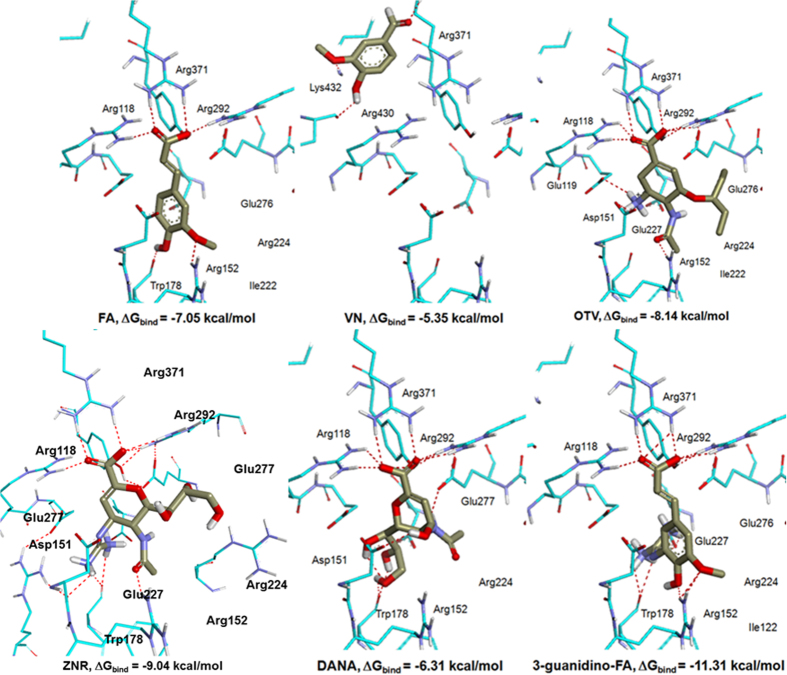
Predicted docking pose of **FA**, **VN** and **3-guanidino-FA** in comparison with the existing inhibitors **OTV, ZNR** and **DANA** onto the NA active site (PDB ID: 3TI6).

We next performed molecular docking of **FA**, **VN** and **3-guanidino-FA** along with **OTV**, **ZNR** and **DANA** onto the NA crystal structure in order to understand their neuraminidase inhibition. Figure 3 shows the docking poses of the six ligands. As predicted, introducing a guanidino group at C3 **(3-guanidino-FA)** increased the binding affinity toward NA with the calculated free energy of binding, ΔG_bind_ = −11.31 kcal/mol which is far more favorable compared to its parent compound, **FA** (−7.05 kcal/mol), **OTV** (−8.14 kcal/mol), **ZNR** (−9.04 kcal/mol), and **DANA** (−6.31 kcal/mol). However, there is a limitation of molecular docking, ΔG_bind_ is thus, not the only key point we consider in determining how good the ligand is docked into the enzyme’s active pocket. The molecular interaction with the essential amino acid residues was also considered to verify whether the ligand is docked in a favorable conformation^32^.

Most of these ligands except **VN**, docked with similar conformations by interacting with conserved amino acid residues such as Arg118, Arg152, Arg292, Arg371, Trp178 and Tyr406. However, the predicted pose of **VN** showed that it bound outside of the NA’s active site, but the *O*-carbonyl was still capable of forming a hydrogen bond with Arg371. VN molecule is quite small, thus it would be easily bound in the NA active site. However, the absence of ionizable group in vanillin will result in vanillin to lose interaction with the Arg triad residues. Introduction of a guanidino group promote the interaction of vanillin with the Arg triad residues at the active site through hydrogen bonding or electrostatic interaction as observed in MY21.

Additional H-bond interactions were formed by **OTV** (Glu119 and Asp151), **ZNR** (Asp151 and Glu276), **DANA** (Asp151 and Glu277), and **3-guanidino-FA** (Glu227).

**Figure 4:**
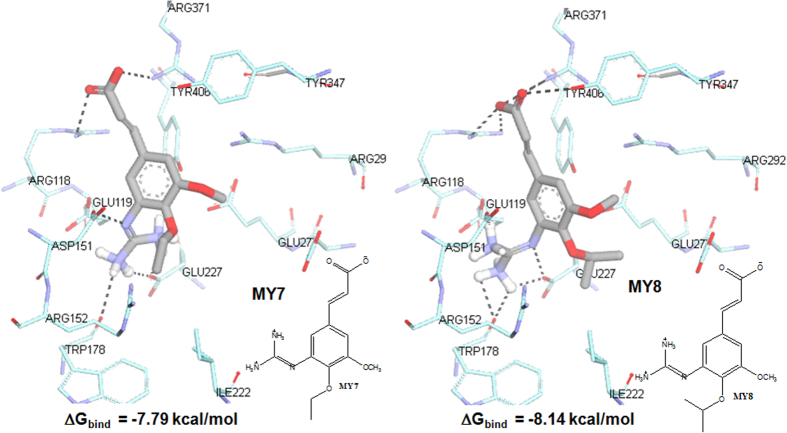
The binding site of **MY7**, Gbind = −7.79 kcal/mol and **MY8**, Gbind = −8.14 kcal/mol, on NA’s active site.

In this study, although **3-guanidino-FA** has the lowest ΔG_bind_, the introduction of a guanidino group at C3 is not strong enough to move the ligand closer to the acidic pocket near 150-loop. Based on the lesson learned from **OTV**’s discovery, increasing the hydrophobic character of **FA** at C2 might further increase the interaction with this 150-loop. Consequently, the hydroxy group at C2 was substituted with two different alkyl groups, i.e. ethyl ether (in compound **MY7**) and isopropyl ether (in **MY8**) with the aim to increase the hydrophobic interaction with the corresponding amino acid residues such as Arg224 and Trp178 (see Fig. 4). This results in a closer proximity of guanidino group at C3 to acidic pocket near 150-loop.

Figure 4 shows the binding of **MY7** and **MY8** to the NA’s active site. Both compounds were observed to have similar binding orientation and formed hydrogen bonds with Arg118, Asp151, Arg152, Glu227, and Arg371. The guanidino group at C3 formed H-bonds with the negatively charged amino acid residues (i.e Asp151 and Glu227,) and an additional H-bond with Arg152. The H-bond angle between the oxygen of the carboxylic acid of the **MY7** and **MY8** and H-N-Arg371 residue deviated about 9 to 13° less than that of **OTV** (118°) resulting in the loss of Arg292 interaction. However, this carboxylate group was still able to interact with Arg118 and Arg371.

In addition, the methoxy (both **MY7** and **MY8**) at C1 position, ethyl ether (**MY7**) and isopropyl ether (**MY8**) at C2 position, shifted closer to Arg224 and Trp178 which may stabilize the surrounding H-bond interactions. Although **MY7** and **MY8** have similar binding modes, replacing the linear alkyl chain with the bulky isopropyl group resulted in a stronger hydrophobic interaction which is reflected in the calculated ΔG_bind_ (**MY7**: −7.79 kcal/mol while **MY8**: −8.14 kcal/mol). The ΔG_bind_ of both **MY7** and **MY8** are lower than **DANA** (−6.31 kcal/mol) but these energies are more comparable with **OTV** (−8.14 kcal/mol) and **ZNR** (−9.04 kcal/mol). Although the loss of interaction with Arg292 decreased the ΔG_bind_, the conformations of **MY7** and **MY8** are more favorable than **DANA** due to their capability to move closer to 150 pocket to interact with Asp151, mimicking the binding mode of **OTV** and **ZNR**^33^.

Unfortunately, the synthesis of **MY7** and **MY8** could not be achieved despite several attempts due to the high impurities present in every single step of the reaction. However, the three intermediate compounds were successfully characterised, i.e. **MY1**–**3** (see Figure S1 of Supporting Information (SI)) and they too were investigated for possible NA inhibition. The intermediate compounds (**MY4**–**6**) were also unsuccessfully isolated. Docking investigation (see Figure S2 of SI) showed that the carboxylate in **MY4** and its ester form in **MY5** dominantly orientated to the arginine triad rather than the acidic pocket. Interestingly, **MY6** could cover almost all residues in the known favorable binding site including the arginine triad, Asp151, Arg152 and Tyr406. The higher ∆G_bind_ (less negative value) of the ligand, the weaker the affinity of the ligand to the active site of the receptor. Here, **MY6** has ∆G_bind_ of −6.43 kcal/mol which is higher than **MY4**–**5** (−8.88 and −8.66 kcal/mol, respectively).

The intermediate compound with the nitro group at C3 position, **MY1,** correlated to 4-(acetylamino)-3-hydroxy-5-nitrobenzoic acid, also known as BANA^34^, which is an aromatic NA inhibitor with a nitro substituent at C3 of benzoic acid scaffold. The contribution of this group was not elucidated but its ionic state could be associated with the optimum pH for ligand-protein binding^35^. The ester group in **MY2** and **MY3** was introduced as a protecting group to the carboxylate group of **MY1**. Closer inspection however, revealed that this could be correlated with **OTV** in its ester form. This provided us the opportunity to modify **MY2** by increasing the alkyl chain length of the ester group started from methyl, ethyl, propyl and butyl-ester; **MY9**–**12,** without the nitro group at C6; **MY13**–**15,** with nitro group at C6 (see Figure S3 of SI). In **MY3**, the isopropyl ether or OCH(CH_3_)_2_ group substituted the OH group at C2. Inspired by the desired compounds, **MY7** and **MY8** which have a hydrophobic group at this position, we attempted to replace the OH group with diverse substituted benzenesulfonyloxy groups to increase the hydrophobic character of the compounds (**MY16**–**20**). Table 1 showed the structure of **MY1**–**23** and the corresponding free energy of binding predicted by molecular docking.

**Figure 5:**
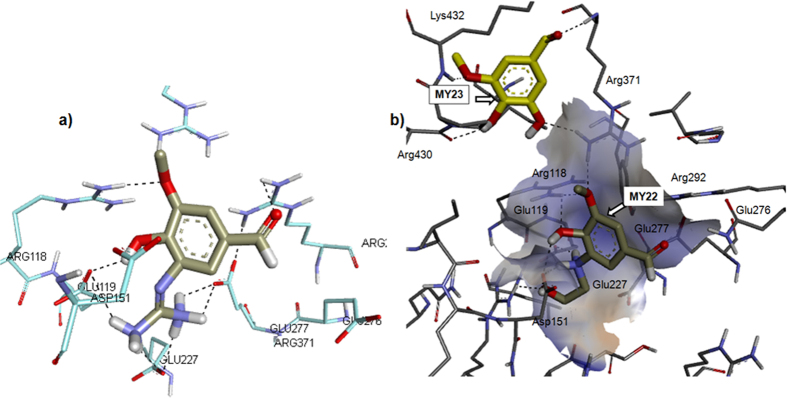
The docked poses of (**a**) **MY21** and (**b**) **MY22** and **MY23** onto NA’s active site with the carbons colored gray (**MY22**) and yellow (**MY23**).

Vanillin was found active in the NA enzymatic assay (see Table 2). Since the main scaffold of **VN** is similar to **FA**, we also explored the possibilities of vanillin derivatives to be applied as NA inhibitors. Addition guanidino at C3 position yielded compounds **MY21**, (see Figure S4 of SI). Alternative to a guanidino group, ***HBA*** (hydrogen bond acceptor) features could be achieved with OH and aminoethanol group. **MY21** can cover the arginine triad pocket as well as the acidic pocket. In addition, the guanidino group also formed interaction with glutamine residues at positions 227 and 277 which are also essential for the enzyme’s activity (see Fig. 5a).

A hydroxy group is also present in **DANA** and aminoethanol should possess the *HBA*/*HBD* (hydrogen bond donor) character like the guanidino group needed to lock the acidic pocket. The substitution of **VN** with either OH (**MY23**) or aminoethanol (**MY22**) at C3 was rationalised as these two functional groups can work as either *HBA* or *HBD* as guanidino. Additional aminoethanol also allowed **MY22** to be docked into the active site but resulted in the OH group at C3 of **MY23** to flip out from the active site. However, **MY23** was still able to make a contact with Arg371 via the carbonyl group of the corresponding amino acid (see Fig. 5b).

In general, based on the docking results, all models (**MY1**–**23**) were predicted to have moderate to strong inhibitory activities thus provide a good rationale for the compounds to be synthesised and evaluated for their activities *in vitro* (see Table 2).

### H1N1 Neuraminidase Inhibition and Antiviral Assay

In the synthesis, two byproducts were successfully isolated and characterised during the preparation of **MY1.** They were ferulic acid with nitro and amino substituents at C6 and C3 positions, respectively. Here, we include those two byproducts (encoded as **MY24** for the nitro substituent and **MY25** for the amino substituent) and also two starting materials (**FA** and **VN**) for the H1N1 neuraminidase studies.

In total, there were 22 compounds tested against H1N1 NA. Seventeen compounds (**MY1–3**, **MY9–22**) were synthesised in our laboratory, two compounds (**MY24** and **MY25**) were collected as the byproducts, while **FA, VN** and **MY23** (3-hydroxyvanillin) were commercially obtained. The initial screening showed that **MY23** have high inhibition towards the enzyme at a concentration of 125 μg/mL (Figures S5 and S6 of SI) and IC50 of 68 μM (see Table 2).

The starting material, **FA** showed 86% inhibition at 125 μg/mL demonstrated that even without any functional group modification, the scaffold itself was able to inhibit the NA activity. However, due to its small size (low molecular weight), the IC_50_ is considered high, i.e. 140 μM (see Table 2). The nitration of **FA** at C3 (**MY1**) increased slightly the enzyme inhibition (IC_50_ of 127 μM). Incidentally, the reactions yielded two byproducts, i.e., **MY24** and **MY25**. **MY24** is a geometric isomer of **6**-**nitro**-**FA** while **MY25** is the reduced form of **MY1** (**3**-**amino**-**FA**). This isomer apparently decreased the activity significantly as shown by its IC_50_ of 489 μM indicating that the structural configuration also influences the ligand-enzyme interaction. Although the difference between the two isomers was slight, it could significantly change the binding mode toward the enzyme or receptor.

Compounds **MY3**, **MY9** and **MY25** also showed good activities towards the inhibition of H1N1 NA with IC_50_ value of 90, 191 and 147 μM, respectively. **MY3**, which has substitutions of a nitro group at C3, an isopropyl ether at C4 as well as an ethyl ester at C5 position showed the best IC_50_ amongst all the derivatives of **FA**.

In general, vanillin derivatives showed activities in the enzymatic assay better than the ferulic acid derivatives. Two of the three vanillin derivatives showed IC_50_ < 100 μM. Vanillin with guanidino group (**MY21**) attached to C3 position was found to show the best IC_50_ (50 μM) reflecting the vital role of this particular functional group in NA inhibitor as previously demonstrated by the structure of zanamivir.

The estimated experimental binding free energy has been calculated using the formula ΔG = −RTlnIC_50_ (see Table 2). The ΔG_bind_ calculated by docking has no significant different with the estimated experimental ΔG_bind,_ therefore, the activity of compounds against NA was determined properly. The ΔG_bind_ of both calculated and estimated experimental was maintained at range 4–8 kcal/mol and 3–5 kcal/mol, respectively.

Four compounds i.e. **FA**, **VN**, **MY3**, **MY21** and **MY23** that showed the lowest IC_50_ from the neuraminidase inhibition assay were checked for their toxicity on the MDCK cells. Compound **MY15** was also selected and purposely used as the negative control as the IC_50_ of >1 mM in the H1N1 NA the neuraminidase inhibition can be regarded as low or no activity. **MY23** was excluded from the subsequent antiviral assay as it showed a significant reduction in cell viability (CC_50_ = 601 μM).

**Figure 6:**
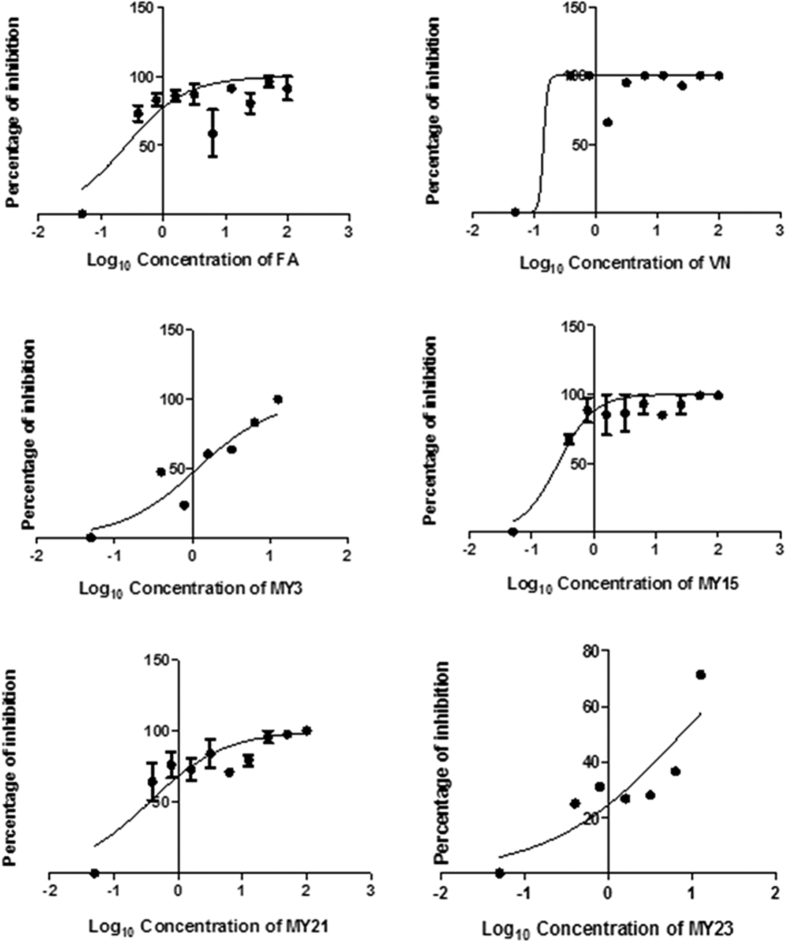
H1N1 viral percentage inhibition versus Log10 concentration of **FA, VN, MY3, MY15, MY21** and **MY23**. The graph was generated using GraphPad Prism 5.01 (www.graphpad.com).

Half maximal effective concentration (EC_50_) was determined from the plot of inhibition percentage against Log_10_ concentration (see Fig. 6). The ratio of CC_50_/EC_50_ was used to analyse the compound’s selectivity. From the results, six compounds showed significant antiviral activities in the MDCK cells. Table 3 presented the CC_50_, EC_50_ and SI of the five selected compounds. **FA**, **VN**, **MY3**, **MY15**, **MY21** exhibited low EC_50_ values (EC_50_ = 1.32 ± 0.08 μM, 6.05 ± 4.3 μM, 7.50 ± 9.3 μM, 0.95 ± 0.02 μM, and 1.70 ± 0.02 μM respectively) without showing any signs of toxicity at high concentrations. The relatively high selectivity index values of these compounds also indicated that they are effective to inhibit the virus growth without affecting the living host cells. Although **MY15** was intended to be used as a negative control, it was surprising to see that it showed quite a high activity toward the virus. Thus, it is suspected that its molecular mechanism to inhibit the virus might be through mechanism other than NA inhibition or through a mixed mechanism that also include NA inhibition.

## Conclusions

We designed two ferulic acid derivatives as novel H1N1 NA inhibitor models mimicking the features of oseltamivir carboxylate. A guanidino group and two different alkyl ether groups were introduced and substituted at C3 and C2 position of ferulic acid, respectively. Docking simulation showed that these two compounds **MY7** and **MY8** demonstrated attractive molecular interactions with the acidic residues near 150-loop compared to the parent compound, ferulic acid. Unfortunately, we unsuccessfully collected these two models in pure compounds when we tried to synthesise them. However, we collected all intermediates to be studied its *in silico* and *in vitro* activity against H1N1 NA. Beside, we also designed some other ferulic acid and vanillin derivatives as H1N1 NA inhibitors followed by synthesis and NA inhibition assay. The study reveals the activity of some FA and VN derivatives against NA at less than 100 μM of IC_50_ and the best activity (IC_50_ = 50 μM) was shown by MY21 (guanidino vanillin) proving the concept of our modeling that introducing guanidine group will enhance the activity as NA inhibitor. Inhibition to the viral replications by some low IC_50_ compounds against NA (FA, VN, MY3, MY15, MY21, and MY23) demonstrated EC_50_ values in the range of 1–41 μM. Therefore, we believe these two models along with the other active ferulic acid and vanillin derivatives, might be a suitable starting compounds for further lead optimization as NA inhibitors.

## Methods

### Molecular Modelling

The control docking was carried out by redocking **OTV** onto its co-complex NA (PDB ID 3TI6). The complex structure was processed using AutoDockTools 4.2 (www.autodock.scripps.edu) with the ligand, Ca ion and water separated from the protein^36^. The protein was protonated with polar hydrogens and given by Kollman charge, while the OTV ligand was protonated with all hydrogens and given by Gasteiger charge. The docking runs were set up with a grid of 60 points each in the x, y and z directions and spacing of 0.375 Å. The mass of the ligand was set to x = −28.597, y = 14.260, z = 21.109 and centered in the NA active site. Genetic Algorithm was chosen for docking calculation in AutoDock 4.2.3^37^ with Ubuntu 12.04 as the operating system. The searching parameters were set to the default values (Population size = 150, maximum number of evaluations = 2500000, maximum of generations = 27000, maximum number of top individuals that automatically survives = 1). The number of GA runs was set to 250. Docking parameters such as random number generation, energy parameters, and step size were also set to the default values. The results were evaluated by analysing the RMSD values, ligand-protein interactions, free energy of binding (ΔG_bind_) as well as the number of conformations exist in a population cluster. The estimated ΔG_bind_ was calculated as the sum of final intermolecular energy, van der Waals, hydrogen bond and desolvation energies, electrostatic energy, final total internal energy, torsional free energy and unbound system’s energy. For the subsequent molecular dockings, **FA** and **VN** derivatives were sketched and their 3D conformations were generated using Hyperchem Professional version 8.0 (www.hyper.com) with MM + force field and Polak-Ribiere (Conjugate Gradient). The visualization of ligand-protein interaction was aided using Discovery Studio 3.5 (www.accelrys.com).

### Synthesis

The synthesis of **FA** derivatives involved four types of reactions, i.e. nitration, esterification, *O*-alkylation and *O*-benzenesulfonylation (see Figure S3 of SI). The steps of the **VN** conversion included aminoethanol substitution, nitro reduction and the conversion of amino to guanidino group were outlined in Figure S4 of SI. The detailed procedure of the synthesis is provided in the Supporting Information. The identities of the compounds synthesized were confirmed by Fourier Transform infrared spectra, mass spectrometry as well as ^1^H and ^13^C-NMR spectra (see Supporting Information). The numbering system of NMR characterization was presented in Table S1 of SI.

### H1N1 Neuraminidase Inhibition and Antiviral Assay

The H1N1 NA assay followed the general procedure of Fluorometric Neuraminidase Assay^38^. The H1N1 NA enzyme was purchased from Sinobio. The fixed concentrations of H1N1 NA (0.3 u/mL) and MUNANA (100 mM) were optimised following the method previously described. DANA and OTV were used as the positive control inhibitors (The assay data is provided in the Figure S6 of SI). H1N1 neuraminidase assay was carried out by preparing the reaction mixture containing an assay buffer, tested samples (at concentrations 7.813 to 250 μg/mL in 2.5% of DMSO-Buffer), and a constant 0.3 unit of neuraminidase were pre-incubated at 37 °C for 30 minutes with 200 rpm. Then after the addition of 100 μM of substrates, the reaction assays were incubated at 37 °C for 60 minutes with 200 rpm. To stop the reaction, 100 μl of glycine stop solution was added. The assays were carried out in triplicate. The fluorescence intensity of NANA was measured by Modulus Microplate Reader with a UV optical kit at λ 340/440 nm.

Madin Darby Canine Kidney (MDCK) epithelial cell line was used for the testing of H1N1 NA inhibitors^39^. To evaluate the cell cytotoxicity of each compound, the cell proliferation assay was carried out using WST-1 reagent. The H1N1 virus used was The A/Malaysia/Muar/33/2009 (H1N1). All reagents for antiviral assay were purchased from Medigene, Sigma, Roche and Bio-Diagnostic. Oseltamivir carboxylate purchased from Toronto Research Chemical Inc. was used as the positive control. Virus titer for H1N1 virus was determined by the plaque reduction assay^40^. Similar to the cytotoxicity study, the cells were seeded in a 24-well plate and incubated overnight at 37 °C in 5% CO_2_. Then, the virus was adsorbed onto the cells for 1 hour at varying concentrations (10- fold dilutions). After incubation, the virus solution was replaced by an overlay medium (carboxymethyl cellulose, CMC) and each plate was incubated for 3 to 5 days until viral plaques were fully formed. Crystal violet was used to stain the cells at the end of the assay, and the plaques in each well were counted and their viral titer determined. The virus dilution that produced ~100 PFU/mL was chosen for the inhibition test.

## Additional Information

**How to cite this article**: Hariono, M. *et al*. Potential New H1N1 Neuraminidase Inhibitors from Ferulic Acid and Vanillin: Molecular Modelling, Synthesis and in Vitro Assay. *Sci. Rep.* **6**, 38692; doi: 10.1038/srep38692 (2016).

**Publisher's note:** Springer Nature remains neutral with regard to jurisdictional claims in published maps and institutional affiliations.

## Supplementary Material

Supplementary Information

## Figures and Tables

**Table 1:** List of ΔG_bind_ of **FA**, **VN** and **MY1**-**23** as calculated using molecular docking.

Ligand	ΔG_bind_ (kcal/mol)	Ligand	ΔG_bind_ (kcal/mol)	Ligand	ΔG_bind_ (kcal/mol)
**MY1**	−7.07	**MY10**	−5.96	**MY18**	−7.77
**MY2**	−6.63	**MY11**	−6.23	**MY19**	−7.90
**MY3**	−7.31	**MY12**	−6.25	**MY20**	−8.50
**MY4**	−8.88	**MY13**	−6.44	**MY21**	−6.09
**MY5**	−8.66	**MY14**	−6.70	**MY22**	−5.87
**MY6**	−6.43	**MY15**	−7.03	**MY23**	−5.42
**MY7**	−7.79	**MY16**	−7.32	**FA**	−7.05
**MY8**	−8.14	**MY17**	−7.45	**VN**	−5.35
**MY9**	−6.01				

**Table 2:**
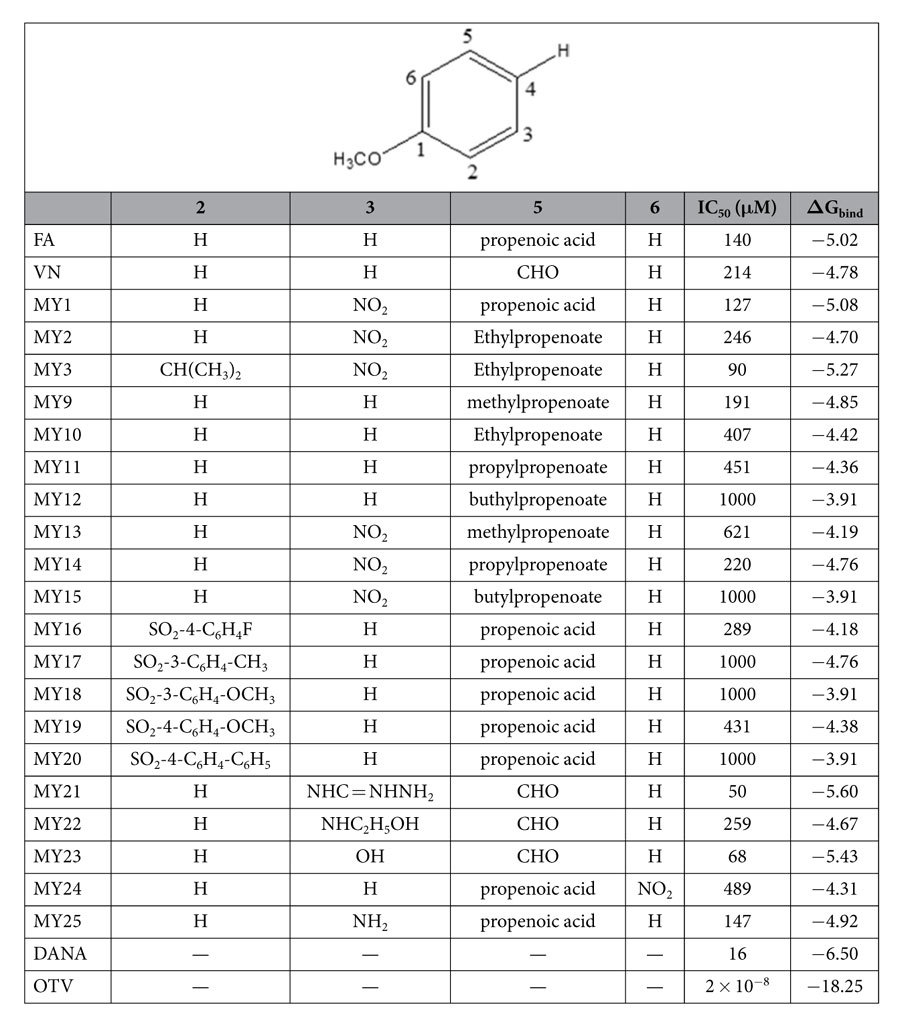
The IC_50_ and estimated experimental ΔG_bind_ values of ferulic acid, vanillin and their derivatives against H1N1 NA.

**Table 3:** The CC_50_, EC_50_ and SI values of ferulic acid, vanillin and their selected derivatives against H1N1 virus.

H1N1 NA Inhibitors	CC_50_ (μM)	EC_50_ (μM)	SI
**FA**	702	1.32 ± 0.08	532
**VN**	657*	6.05 ± 4.3	109
**MY3**	321*	7.50 ± 9.3	43
**MY15**	352*	0.95 ± 0.02	373
**MY21**	478*	1.70 ± 0.02	281
**MY23**	601	40.46 ± 3.7	15
Oseltamivir carboxylate	351*	34.956	10

SI = Safety Index was generated by the ratio of CC_50_ and EC_50_.

^*^Minimum toxic concentration.
