# Supplementary material for: Potential New H1N1 Neuraminidase Inhibitors from Ferulic Acid and Vanillin: Molecular Modelling, Synthesis and in Vitro Assay
Source: Sci Rep. 2016 Dec 20;6:38692. doi: 10.1038/srep38692 (PMC5171792; doi:10.1038/srep38692)
Supplement: Supplementary Information [file srep38692-s1.pdf]

Supporting Information

Potential New H1N1 Neuraminidase Inhibitors from Ferulic Acid and Vanillin: Molecular Modelling, Synthesis and in Vitro Assay

Maywan Hariono[1], Nurshariza Abdullah[2], KV Damodaran[1,+], Ezatul E. Kamarulzaman[1,+], Nornisah Mohamed[1,+], Sharifah Syed Hassan[3,+], Shaharum Shamsuddin[2,+], and Habibah A. Wahab[1,4,*]

[1]School of Pharmaceutical Sciences, Universiti Sains Malaysia, 11800 Minden, Pulau Pinang, Malaysia

[2]School of Health Sciences, Universiti Sains Malaysia, 16150 Kubang Krian, Kelantan, Malaysia

[3]Jeffry Cheah School of Medicine and Health Sciences, Monash University Malaysia, 47500 Bandar Sunway, Selangor Darul Islam, Malaysia

[4]Malaysian Institute of Pharmaceuticals and Nutraceuticals, Ministry of Science, Technology and Innovation, Halaman Bukit Gambir, 11900 Bayan Lepas, Pulau Pinang, Malaysia

Keywords: H1N1; Neuraminidase inhibitor; Ferulic acid; *In vitro* assay; Antiviral assay; QSAR; Molecular docking

***Corresponding author emails:** habibahw@usm.my; habibah@ipharm.gov.my

***Corresponding author phone:** H. Wahab:604-6533 ext 2206, 2238, fax: 604-6570017

Synthesis of ferulic acid and vanillin derivatives

(*E*)-3-(4-hydroxy-3-methoxy-5-nitrophenyl)acrylic acid (**MY1**)

The synthesis of **MY1** followed the general procedure for nitration[1]. Briefly, **FA** (41 mmol) was dissolved into 58 mL of glacial acetic acid while warmed up, and then cooled down at a room temperature. Fuming nitric acid (1.45 mL) was carefully added into the cool solution, and stirred up over a period of 60 minutes to get a brown mixture. The mixture was dropped gently into a bulk volume of ice water until the yellow solid precipitated out. These were filtered, washed with water and allowed to dry and then purified using column chromatography. Yellow powder; yield 82%; m. p. 230-233°C; υ_{max}/ cm^{-1}(KBr) 3354 (OH), 1618 (C=O); ^{1}H-NMR (DMSO-d_6) δ 3.91 (1H, s, H7), 3.94 (3H, *s*, H9), 6.60 (1H, *d*, J_{trans}= 16 Hz, H11), 7.57 (1H, *d*, J_{trans}= 16 Hz, H10), 7.64 (1H, *d*, J_{meta} = 1.5 Hz, H6), 7.75 (1H, *d*, J_{meta}= 1.5 Hz, H4), 12.34 (1H, *br, s*, H13); δ_C 56.1 (C9), 111.5 (C4), 115.9 (C11), 116.1 (C6), 123.2 (C5), 126.2 (C3), 144.9 (C2), 148.3 (C10), 149.4 (C1), 168.5 (C12); QTOF-MS m/z calcd for $C_{10}H_{13}N_2O_6$ $[M+NH_4]^+$ 257.2200, found 257.2999.

(*E*)-ethyl 3-(4-hydroxy-3-methoxy-5-nitrophenyl)acrylate(**MY2**)*

The synthesis of **MY2** followed the general procedure for esterification[2]. **MY1** (4.18 mmol) was dissolved into the corresponding alcohol (8 mL) while cooling down the rounded bottom flask into the ice water. A concentrated H_2SO_4 was added carefully into the cool solution and allowed to reflux at 60-70°C for 24 hours. The mixture was neutralized with 25 mL of $NaHCO_3$ 10% and then extracted using ethyl acetate (2 x 25 mL) followed by washing it with 2 x 25 mL of water. The ethyl acetate was dried over anhydrous $MgSO_4$ and then concentrated under reduced pressure. The crude product was subjected to the preparative thin layer chromatography (Silica gel, *n*-hexane: EtOAc (2:2)) to afford the ester

product.Yellowpowder; yield ; m.p. 144-147°C; υ_{max}/ cm^{-1} (KBr), 3465 (OH), 1614 (C=C), 1536 (C=O); ^{1}H-NMR ($CDCl_3$) δ 1.27 (3H, *t*, *J* = 7 Hz, H15), 3.91 (3H, *s*, H8), 4.21 (2H, *q*, *J* = 8 Hz, H14), 6.32 (1H, *d*, J_{trans} = 16 Hz, H11), 7.19 (1H, *s*, H6), 7.52 (1H, *d*, J_{trans} = 16 Hz, H10), 7.79 (1H, *s,* H4), 10.85 (1H, *br, s*, H9);δ_C 14.6 (C15), 57.2 (C14), 60.4 (C8), 113.9 (C4), 117.9 (C11), 118.3 (C6), 137.7 (C5), 143.4 (C3), 150.4 (C2), 152.2 (C1), 166.7 (C12).

(*E*)-ethyl 3-(4-isopropoxy-3-methoxy-5-nitrophenyl)acrylate (**MY3**)

The synthesis of **MY3** followed the general procedure for alkylation[3]. A mixture containing the corresponding alkyl bromide (1.315 mmol), *tetra*-butylammonium iodide (TBAI) (equal to the corresponding number of alkylation) in 3.5 mL of DMF was freshly prepared. In a separate flask, **MY2** (0.598 mmol) was mixed with sodium carbonate (Na_2CO_3) (equal to the corresponding number of alkylation) in 3.5 mL of dimethylformamide (DMF) and then stirred vigorously for 15 minutes. The first mixture was added into the second mixture and the stirring was continued at room temperature for six hours. The reaction progress was monitored by TLC using *n*-hexane: ethyl acetate (EtOAc) (1:3) as a mobile phase. After the product formed, the reaction mixture was diluted with 70 mL of water and then extracted using 3 x 70 mL of EtOAc. The organic phase was collected, washed with 3 x 70 mL of water and then dried over anhydrous magnesium sulfate. This organic phase was then evaporated *in vacuo* and then purified using PLC with the same mobile phase system used in the monitoring of the reaction progress. Dark brown powder; yield 9%; m.p. 95-100°C; υ_{max}/ cm^{-1} (KBr), 3436 (OH), 1597 (C=O); ^{1}H-NMR ($CDCl_3$) δ 1.18 (3H, *t*, *J* = 7 Hz, H17), 1.23 (6H, *d*, *J* = 7 Hz, H9 and H20), 3.67 (3H, *s*, H11), 4.13 (2H, *q*, *J* = 8 Hz, H16), 4.13-4.16 (1H, *m*, H8), 6.06 (1H, *d*, J_{trans} = 16 Hz, H13), 6.82 (1H, *s*, H6), 7.40 (1H, *d*, J_{trans} = 16 Hz, H12), 7.59 (1H, *d*, J_{meta} = 1.5, H4); QTOF-MS m/z calcd for $C_{15}H_{20}NO_6$ $[M+H]^+$ 310.3224, found 310.3345.

(*E*)-methyl 3-(4-hydroxy-3-methoxyphenyl)acrylate (**MY9**)

The synthesis of MY9 adopted the procedure of synthesis **MY2** by substituting **MY1** and ethanol with **FA** and methanol, respectively.Colorless amorphous; yield 61%; υ_{max}/ cm^{-1}, 3404 (OH), 1701 (C=O) ; ^{1}H-NMR ($CDCl_3$) δ 3.79 (3H, *s*, H8), 3.92 (3H, *s*, H14), 5.89 (1H, *s*, H9), 6.29 (1H, *d*, J_{trans} = 16 Hz, H11), 6.91 (1H, *d*, J_{ortho} = 8.5 Hz, H3), 7.02 (1H, *d*, J_{meta} = 2 Hz, H6), 7.06 (1H, *d*, J_{meta} = 2 Hz, H4), 7.62 (1H, *d*, J_{trans} = 16 Hz, H10); δ_C 51.69 (C14), 56.16 (C8), 111.75 (C6), 114.62 (C3), 115.97 (C11), 123.58 (C4), 125.96 (C5), 145.57 (C10), 148.39 (C2), 149.98 (C1), 167.56 (C12); QTOF-MS m/z calcd for $C_{11}H_{13}O_4[M+H]^+$ 209.2185, found 209.0179.

(*E*)-ethyl 3-(4-hydroxy-3-methoxyphenyl)acrylate (**MY10**)*

The synthesis of **MY10** adopted the procedure of synthesis **MY9** by substituting methanol with ethanol. Colorless amorphous; yield 68%; υ_{max}/ cm^{-1},3442 (OH), 1605 (C=O); ^{1}H-NMR ($CDCl_3$) δ 1.33 (3H, *t*, *J* = 7 Hz, H15), 3.92 (3H, *s*, H8), 4.25 (2H, *q*, *J* = 7 Hz, H14), 5.85 (1H, *s*, H9), 6.28 (1H, *d*, J_{trans} = 16.5 Hz, H11), 6.91 (1H, *d*, J_{ortho} = 8.5 Hz, H3), 7.03 (1H, *d*, J_{meta} = 2 Hz, H6), 7.07 (1H, *dd*, J_{ortho} = 9 Hz, J_{meta}= 2.5 Hz, H4), 7.61 (1H, *d*, J_{trans}= 16 Hz, H10); δ_C 14.7 (C15), 56.1 (C8), 60.1 (C14), 111.6 (C6), 115.0 (C3), 115.9 (C11), 123.5 (C4), 126.0 (C5), 145.3 (C2), 148.3 (C10), 149.7 (C1), 167.0 (C12).

(*E*)-propyl 3-(4-hydroxy-3-methoxyphenyl)acrylate (**MY11**)*

The synthesis of **MY11** adopted the procedure of synthesis **MY9** by substituting methanol with *n*-propanol. Colorless amorphous; yield 53%; υ_{max}/ cm^{-1}, 3434 (OH), 1605(C=O); ^{1}H-NMR ($CDCl_3$) δ 0.89 (2H, *m*, H15), 0.99 (3H, *t*, *J* = 7.5 Hz, H16), 3.92 (3H, *s*, H8), 4.15 (2H, *t*, *J* = 7 Hz, H14), 5.85 (1H, *s*, H9), 6.29 (1H, *d*, J_{trans}= 16 Hz, H11), 6.91 (1H, *d*, J_{ortho}= 8.5 Hz, H3), 7.03 (1H, *d*, J_{meta}= 2 Hz, H6), 7.07 (1H, *dd*, J_{ortho} = 8 Hz, J_{meta} = 1.5 Hz, H4), 7.61 (1H, *d*, J_{trans}= 16 Hz, H10); δ_C 10.8 (C16), 22.1 (C15),56.1 (C8), 65.6

(C14), , 111.6 (C6), 114.9 (C11), 115.9 (C3), 123.6 (C4), 126.05 (C5), 145.4 (C2), 148.3 (C10), 149.7 (C1), 167.1 (C12).

(*E*)-butyl 3-(4-hydroxy-3-methoxyphenyl)acrylate (**MY12**)*

The synthesis of **MY12** adopted the procedure of synthesis **MY9** by substituting methanol with *n*-butanol. Brown amorphous; yield 38%; υ_{max}/ cm^{-1}, 3438 (OH), 1605(C=O); ^{1}H-NMR ($CDCl_3$) δ 0.96 (3H, *t*, *J* = 7.5 Hz, H17), 1.44 (2H, *m*, H16), 1.69 (2H, *m*, H15), 3.92 (1H, *s*, H8), 4.20 (2H, *t*, *J* = 6.5 Hz, H14), 5.92 (1H, *s*, H9), 6.28 (1H, *d*, J_{trans} = 16 Hz, H11), 6.91 (1H, *d*, J_{ortho} = 8 Hz, H3), 7.03 (1H, *d*, J_{meta}= 2 Hz, H6), 7.06 (1H, *dd*, J_{ortho} = 8 Hz, J_{meta} = 1.5 Hz, H4), 7.61 (1H, *d*, J_{trans} = 16 Hz, H10); δ_C 14.0 (C17), 19.1 (C16),30.8 (C15) 56.1 (C8), 63.8 (C14), 111.6 (C6), 114.9 (C11), 115.9 (C3), 123.6 (C4), 126.0 (C5), 145.4 (C2), 148.3 (C10), 149.7 (C1), 167.2 (C12).

(*E*)-methyl 3-(4-hydroxy-3-methoxy-5-nitrophenyl)acrylate (**MY13**)*

The synthesis of **MY13** adopted the procedure of synthesis **MY2** by substituting ethanol with methanol. Yellow powder; yield 18%; m. p. 165-169°C; υ_{max}/ cm^{-1} (KBr), 3424 (OH), 1716 (C=O); ^{1}H-NMR ($CDCl_3$) δ 3.82 (3H, *s*, H14), 3.99 (3H, *s*, H8), 5.51 (1H, br, *s*, H9), 6.39 (1H, *d*, J_{trans} = 16 Hz, H11), 7.20 (1H, *d*, J_{meta}= 1.5 Hz, H6), 7.60 (1H, *d*, J_{trans}= 16.5 Hz, H10), 7.86 (1H, *d*, J_{meta} = 1.5 Hz, H4); δ_C 51.8 (C14), 57.1 (C8), 110.5 (C4), 117.1 (C6), 122.1 (C11), 123.7 (C5), 132.8 (C3), 137.5 (C2), 140.5 (C10), 143.7 (C1), 167.2 (C12).

(*E*)-propyl 3-(4-hydroxy-3-methoxy-5-nitrophenyl)acrylate (**MY14**)*

The synthesis of **MY14** adopted the procedure of synthesis **MY2** by substituting ethanol with *n*-propanol.Yellow powder; yield 67%; m. p. 80-83°C; υ_{max}/ cm^{-1} (KBr), 3428 (OH), 1699 (C=O); ^{1}H-NMR ($CDCl_3$) δ 1.00 (3H, *t*, *J* = 7.5 Hz, H16), 1.73 (2H, *m*, H15),

3.99 (3H, *s*, H8), 4.18 (2H, *t*, *J* = 6.5 Hz, H14), 6.39 (1H, *d*, J_{trans}= 15 Hz, H11), 7.27 (1H, *d*, J_{meta}= 2 Hz, H6), 7.59 (1H, *d*, J_{trans} = 15 Hz, H10), 7.86 (1H, *d*, J_{meta}= 1.5 Hz, H4), 10.92 (1H, *br*, *s*, H9); δ_C 10.8 (C16), 22.1 (C15),57.3 (C8), 65.9 (C14), 114.1 (C4), 118.2 (C11), 125.1 (C6),130.0 (C5), 137.7 (C3), 137.2 (C2) 143.3 (C10), 150.2 (C1), 166.7 (C12).

(*E*)-butyl 3-(4-hydroxy-3-methoxy-5-nitrophenyl)acrylate (**MY15**)*

The synthesis of **MY15** adopted the procedure of synthesis **MY2** by substituting ethanol with *n*-buthanol. Yellow powder; yield 33% ; m.p. 97-100°C; υ_{max}/ cm^{-1} (KBr), 3448 (OH), 1712 (C=O); ^{1}H-NMR ($CDCl_3$) δ 0.97 (3H, *t*, *J* = 7 Hz, H17), 1.45 (2H, *m*, H16), 1.69 (2H, *m*, H15), 3.99 (3H, *s*, H8), 4.22 (2H, *t*, *J* = 7 Hz, H14), 6.39 (1H, *d*, J_{trans} = 16 Hz, H11), 7.27 (1H, *d*, J_{meta}= 1.5 Hz, H6), 7.58 (1H, *d*, J_{trans} = 16 Hz, H10), 7.86 (1H, *d*, J_{meta} = 1.5 Hz, H4), 10.92 (1H, *br*, *s*, H9); δ_C 14.0 (C17), 19.1 (C16),30.7 (C15) 57.3 (C8), 64.1 (C14), 114.2 (C4), 118.1 (C11), 118.2 (C6), 125.2 (C5), 137.7 (C3), 143.3 (C2), 144.6 (C10), 150.2 (C1), 166.7 (C12).

(*E*)-3-(4-(4-fluorobenzenesulfonoyl)-3-methoxyphenyl)acrylic acid (**MY16**)

The synthesis of **MY16** followed the general procedure for tosylation[4]. **FA** (2.57 mmol) was dissolved in 3 mL of pyridine at 0°C and then dropped carefully by 4-fluorobenzene-1-sulfonyl chloride. The mixture was stirred over a period of 2 hours until the white precipitates were coming and then neutralized using HCl 10%. The precipitates were then filtered, washed with water and recrystallised from chloroform to afford the pure product.Yield 24%; m. p. 149-151°C; υ_{max}/ cm^{-1} (KBr) 3432 (OH), 1691 (C=O); ^{1}H-NMR ($CDCl_3$) δ 3.94 (3H, *s*, H8), 6.29 (1H, *d*, J_{trans}= 16, H21), 6.93 (1H, *d*, J_{ortho} = 8 Hz, H6), 7.58 (1H, *d*, J_{meta} = 1.5, H3), 7.11-7.24 (4H, *m*, H15, H11, H14, H12), 7.70 (1H, *d*, J_{trans}= 16 Hz, H20), 7.90 (1H, *dd*, J_{ortho} = 8.5, J_{meta}= 2.5, H4); δ_C 56.2 (C8), 112.3 (C6), 112.9 (C21), 121.5

(C3), 124.1 (C13), 125.8 (C12), 128.6 (C14), 129.7 (C4), 135.1 (C5), 135.9 (C11), 139.0 (C15), 139.9 (C2), 143.1 (C10), 152.0 (C20), 180.6 (C22);); QTOF-MS m/z calcd for $C_{18}H_{16}FNO_6S$ $[M+ACN]^+$393.3861, found 393.2523.

(*E*)-3-(4-(3-methylbenzenesulfonoyl)-3-methoxyphenyl)acrylic acid (**MY17**)*

The synthesis of **MY17** adopted the procedure of synthesis **MY16** by substituting 4-fluorobenzene-1-sulfonyl chloride with 3-methylbenzene-1-sulfonyl chloride. White powder; Yield 14%; m. p. >300^oC; υ_{max}/ cm^{-1} (KBr) 3428 (OH), 1687 (C=O); ^{1}H-NMR ($CDCl_3$) δ 2.43 (3H, *s*, H16), 3.89 (3H, *s*, H8), 6.37 (1H, *d*, J_{trans}= 16, H21), 6.99 – 7.74 (7H, *m*, H3, H6, H4, H14, H13, H11 and H15), 7.86 (1H, *d*, J_{trans}= 16, H20); δ_C 56.4 (C8), 112.3 (C6), 112.9 (C21), 120.8 (C3), 121.0 (C4), 121.5 (C15), 121.6 (C11), 124.0 (C5), 131.2 (C14), 136.6 (C13), 139.5 (C12), 139.6 (C2), 143.1 (C20), 152.1 (C10), 159.9 (C1), 167.8 (C22).

(*E*)-3-(4-(3-methoxybenzenesulfonoyl)-3-methoxyphenyl)acrylic acid (**MY18**)*

The synthesis of **MY18** adopted the procedure of synthesis **MY16** by substituting 4-fluorobenzene-1-sulfonyl chloride with 3-methoxybenzene-1-sulfonyl chloride. White powder; Yield 42%; m. p. >300^oC; υ_{max}/ cm^{-1} (KBr) 3416 (OH), 1687 (C=O), 1630 (C=C); ^{1}H-NMR ($CDCl_3$) δ 3.64 (3H, *s*, H8), 3.83 (3H, *s*, H17), 6.38 (1H, *d*, J_{trans}= 16 Hz, H22), 7.00-7.47 (7H, *m*, H3, H6, H4, H13, H14, H11 and H15), 7.67 (1H, *d*, J_{trans}= 16 Hz, H21); δ_C 56.3 (C8), 56.4 (C17), 112.8 (C13 and C6), 115.0 (C22), 121.5 (C3), 124.1 (C11), 124.1 (C4), 126.6 (C5), 131.1 (C14), 139.6 (C2), 141.1 (C21), 143.0 (C10), 145.9 (C1), 152.0 (C12), 164.6 (C23).

(*E*)-3-(4-(4-methoxybenzenesulfonoyl)-3-methoxyphenyl)acrylic acid (**MY19**)*

The synthesis of **MY19** adopted the procedure of synthesis **MY16** by substituting 4-methoxybenzene-1-sulfonyl chloride with 3-methoxybenzene-1-sulfonyl chloride. White powder; Yield 54%; decomp. 280°C; υ_{max}/ cm^{-1} (KBr) 3007 (OH), 1728 (C=O); ^{1}H-NMR ($CDCl_3$) δ 3.88 (3H, *s*, H28), 3.89 (3H, *s*, H17), 6.39 (1H, d, J_{trans}= 16 Hz, H22), 6.96-7.82 (7H, *m*, H3, H6, H4, H14, H12, H15 and H11), 7.67 (1H, *d*, J_{trans}= 16 Hz, H21); δ_C 56.1 (C8), 56.2 (C17), 111.6 (C6), 115.9 (C22, C14 and C12), 116.0 (C3), 123.2 (C4 and C5), 126.2 (C11 and C14), 144.9 (C2), 148.3 (C10), 149.5 (C21), 151.3 (C1), 168.4 (C23).

(*E*)-3-(4-(4-phenoxybenzenesulfonoyl)-3-methoxyphenyl)acrylic acid (**MY20**)*

The synthesis of **MY20** adopted the procedure of synthesis **MY16** by substituting 4-phenoxybenzene-1-sulfonyl chloride with 3-methoxybenzene-1-sulfonyl chloride. White powder; Yield 10%; decomp. 280°C; υ_{max}/ cm^{-1}(KBr) 3060 (OH), 1687 (C=O); ^{1}H-NMR ($CDCl_3$) δ 3.67 (3H, *s*, H8), 6.39 (1H, d, J_{trans}= 16 Hz, H27), 7.05-7.45 (9H, *m*, H3, H6, H4, H13, H14, H11, H12, H18 and H19), 7.69 (1H, *d*, J_{trans}= 16 Hz, H26), 7.81-7.95 (3H, *m*, H21, H19 and H20); δ_C 56.3 (C8), 112.9 (C6), 117.8 (C27), 117.9 (C3), 119.2 (C18 and C19), 120.8 (C12 and C14), 121.6 (C4), 124.0 (C20), 124.3 (C11 and C15), 125.8 (C22 and C20), 127.9 (C5), 139.0 (C2), 143.2 (C10), 145.0 (C26), 152.00 (C1), 154.75 (C17), `162.09 (C13); 167.90 (C28).

2-(5-formyl-2-hydroxy-3-methoxyphenyl)guanidine (**MY21**)

The synthesis of **MY21** followed the general procedure for amino to guanidine conversion[6]. Cyanamide (50.27 mmol) was mixed with water and then **3-aminovanillin** was gradually added in 2.5 mL of HCl 1N. The mixture was then refluxed at 80-90°C for 4 hours. After completed, the mixture was cooled down at a room temperature and followed by filtration.

The formed precipitated was then collected, washed with a cold methanol and then dried up under fume hood and purified using TLC (silica F_{254}; $CHCl_3$- MeOH (1:3)) to afford the final product. Brown powder; yield 48%; m.p. 284-286°C;υ_{max}/ cm^{-1} (KBr), 3203 and 3077 (NH_2), 1630 (C=C), 1548 (C=O); ^{1}H-NMR (DMSO-D_6) δ_H 1.61 (2H, s, H14), 3.66 (3H, s, H9), 6.82 (1H, d, J_{meta} = 2 Hz, H6), 7.96 (1H, d, J_{meta}= 2 Hz, H4), 9.39 (1H, s, H10); δ_C 55.5 (C9), 116.0 (C4 and C6), 135.3 (C5), 156.3 (C3 and C2), 165.4 (C1), 175.1 (C13), 188.3 (C10);); QTOF-MS m/z calcd for $C_9H_{11}N_3O_3$ $[M]^+$ 209.2019, found 209.1438.

3-(2-hydroxyethylamino)-4-hydroxy-5-methoxybenzaldehyde (**MY22**)*

The synthesis of **MY22** followed the general procedure for amino substitution[7]. 5-bromovanillin (9 mmol) was dissolved in DCM while adding 2-ethanolamine (13.5 mmol) and 1.8 mL of DIPEA. The mixture was stirred gently overnight at room temperature until the yellow precipitate formed, followed by filtering them to collect the precipitate as the crude product. The filtrate in DCM was washed using water, 10% Na_2CO_3 to collect the DCM phase and followed by evaporating them *in vacuo* to get the yellow residue. This residue was combined with the earliest precipitate as **MY22**.Yellowish green powder; Yield 52%; m.p. 82-84°C; υ_{max}/ cm^{-1} (KBr), 3326 (OH), 1675 (C=O); ^{1}H-NMR. ($CDCl_3$) δ 2.66 (2H, *t*, *J* = 5.5 Hz, H14), 3.43 (2H, *t*, *J* = 5.5 Hz, H13), 3.73 (3H, *s*, H9), 7.21 (1H, *d*, J_{meta} = 1.5 Hz, H6), 7.59 (1H, *d*, J_{meta}= 2 Hz, H4), 7.97 (1H, *s*, H10); δ_C 43.7 (C13), 56.0 (C9), 61.2 (C14), 109.3 (C6 and C4), 134.7 (C5 and C2), 148.9 (C1), 160.3 (C10).

(*E*)-3-(4-hydroxy-3-methoxy-2-nitrophenyl)acrylic acid (**MY24**).

MY24 was isolated and characterized as the by product of the synthesis of **MY1**. Yellow powder; yield 9%; m.p. 165-169°C; υ_{max}/ cm^{-1} (KBr) 3469 (OH), 1597 (C=O);^{1}H-NMR ($CDCl_3$) δ 3.95 (3H, *s*, H9), 6.03 (1H, *s*, H7), 7.14 (1H, *dd*, J_{ortho}= 10 Hz, $J_{allylic}$ = 1.5

Hz, H3, H4), 7.51 (1H, d, J_{trans}= 14 Hz, H11), 7.95 (1H, d, J_{trans} = 14 Hz, H10); δ_C 56.3 (C9), 112.7 (C11), 116.2 (C4), 122.0 (C3), 126.3 (C5), 135.5 (C6), 140.7 (C2), 148.6 (C10), 151.7 (C12); QTOF-MS m/z calcd for $C_{10}H_9NNaO_6$ $[M+Na]^+$ 262.1707, found 262.1653.

(*E*)-3-(3-amino-4-hydroxy-5-methoxyphenyl)1acrylic acid (**MY25**)

MY25 was isolated and characterized as the by product of the synthesis of **MY1**. Dark brown powder; yield 9%; decomp. 280°C; υ_{max}/ cm^{-1} (KBr), 3444, 3387 (NH_2), 1704 (C=O);^{1}H-NMR (DMSO) δ_H 3.81 (3H, *s*, H9), 6.27 (1H, *d*, J_{trans} = 15.5 Hz, H11), 7.16 (1H, *s*, H4), 7.23 (1H, *d*, J_{trans}= 16 Hz, H10), 7.70 (1H, *s*, H6); QTOF-MS m/z calcd for $C_{10}H_{15}N_2O_4[M+NH_4]^+$227.2371, found 227.2268.

*No mass data of compound due to its solubility problem in the reverse phase system of standard protocol of LC-MS in our laboratory.

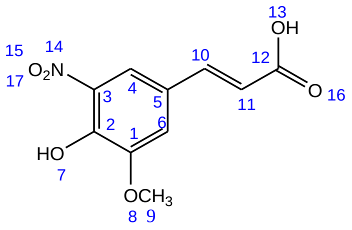 **MY1**	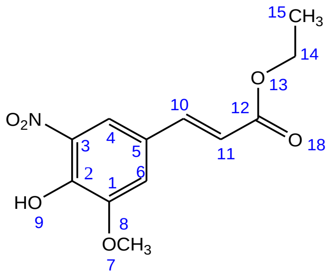 **MY2**	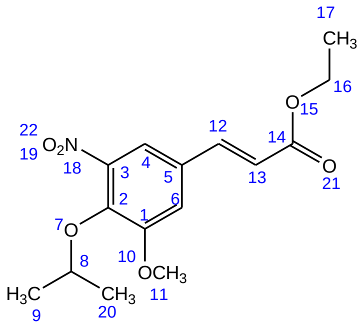 **MY3**
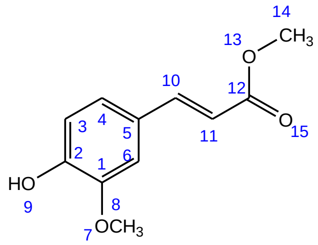 **MY9**	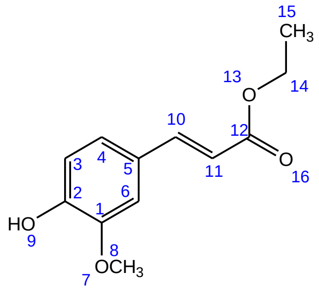 **MY10**	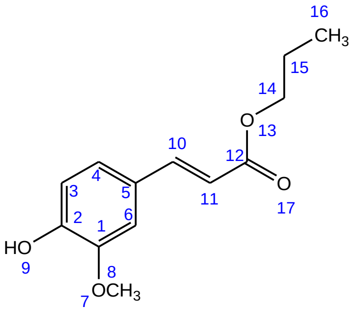 **MY11**
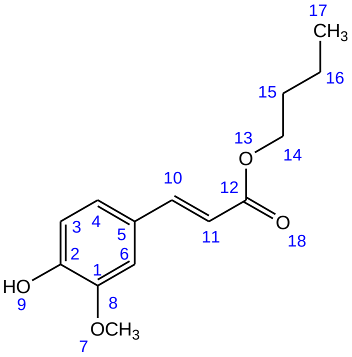 **MY12**	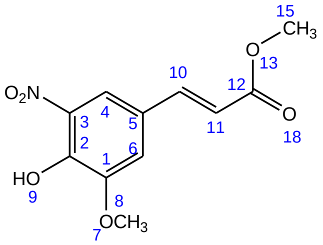 **MY13**	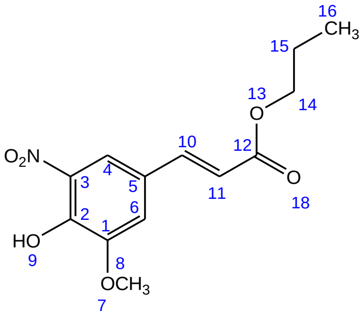 **MY14**

Table S1. Continue.

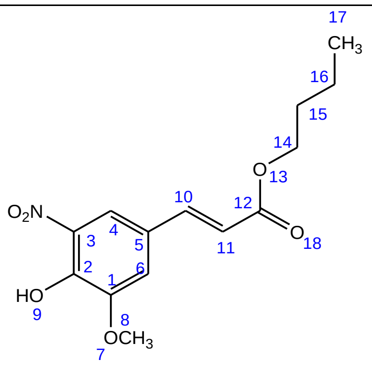 **MY15**	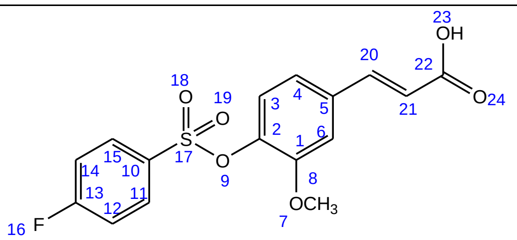 **MY16**
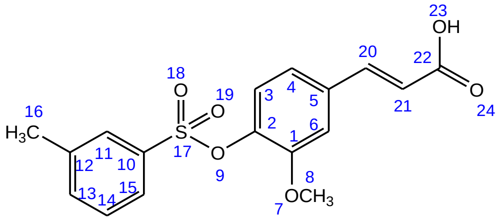 **MY17**	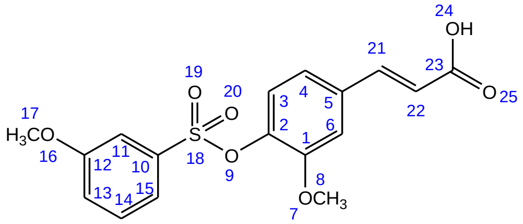 **MY18**
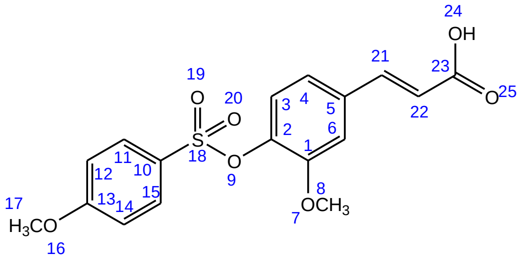 **MY19**	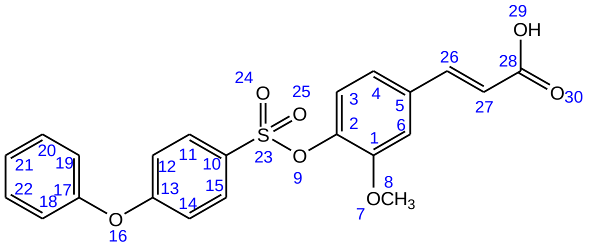 **MY20**
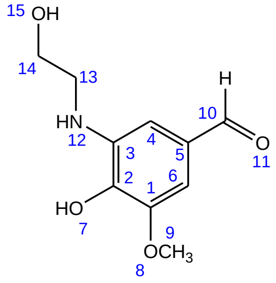 **MY22**	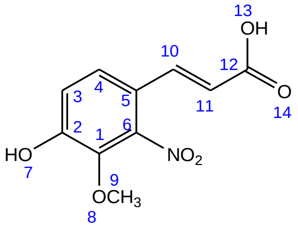 **MY24**

Table S1. Continue.

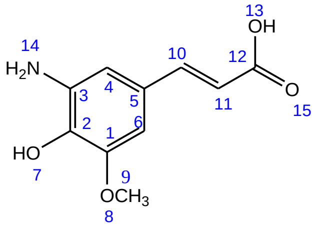

MY25

Table S1. Structure of **MY1-3**, **MY9-20**, **MY22**, **MY24 and MY25**. The numbering system is according to the NMR characterization.

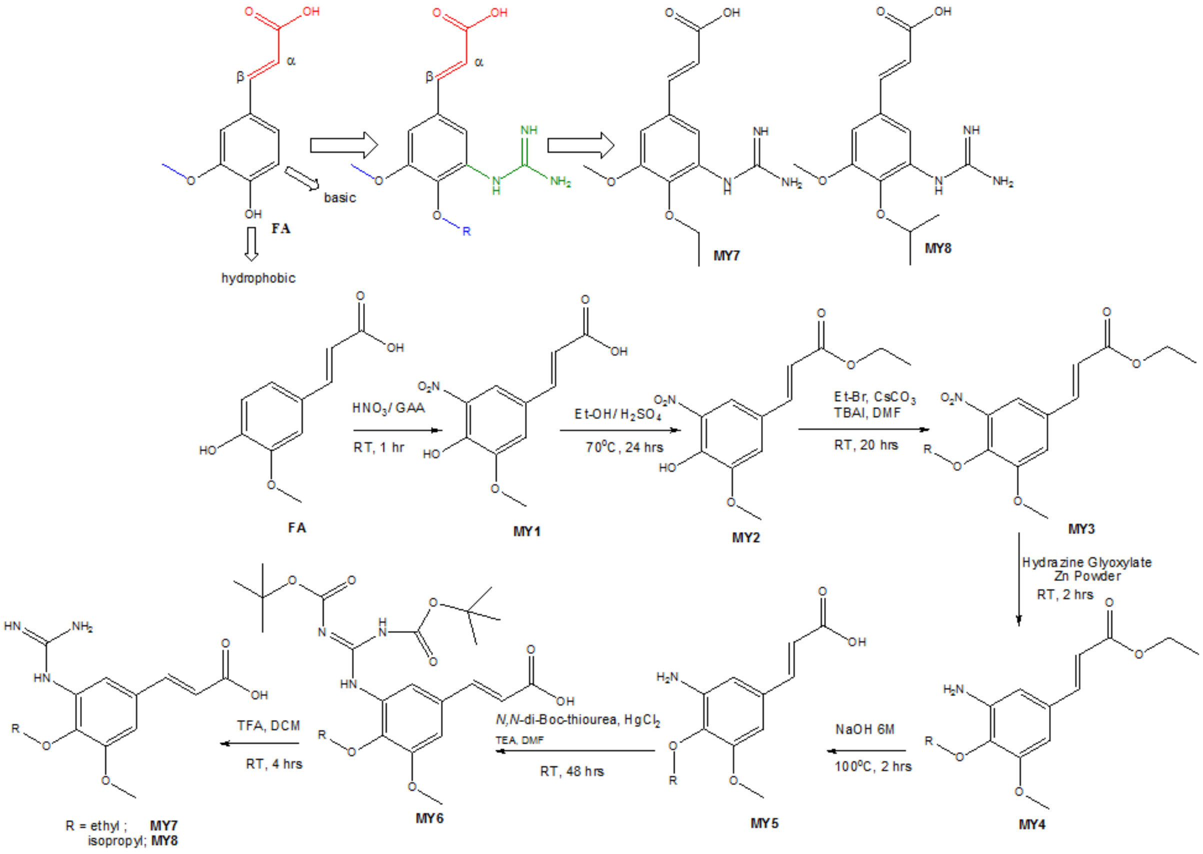

Figure S1. Incorporation of guanidine and alkyl ether groups in ferulic acid scaffold and the retrosynthesis scheme of **MY7** and **MY8.** The structures were drawn using ChemDraw Ultra 8.0 (www.chembridgesoft.com).

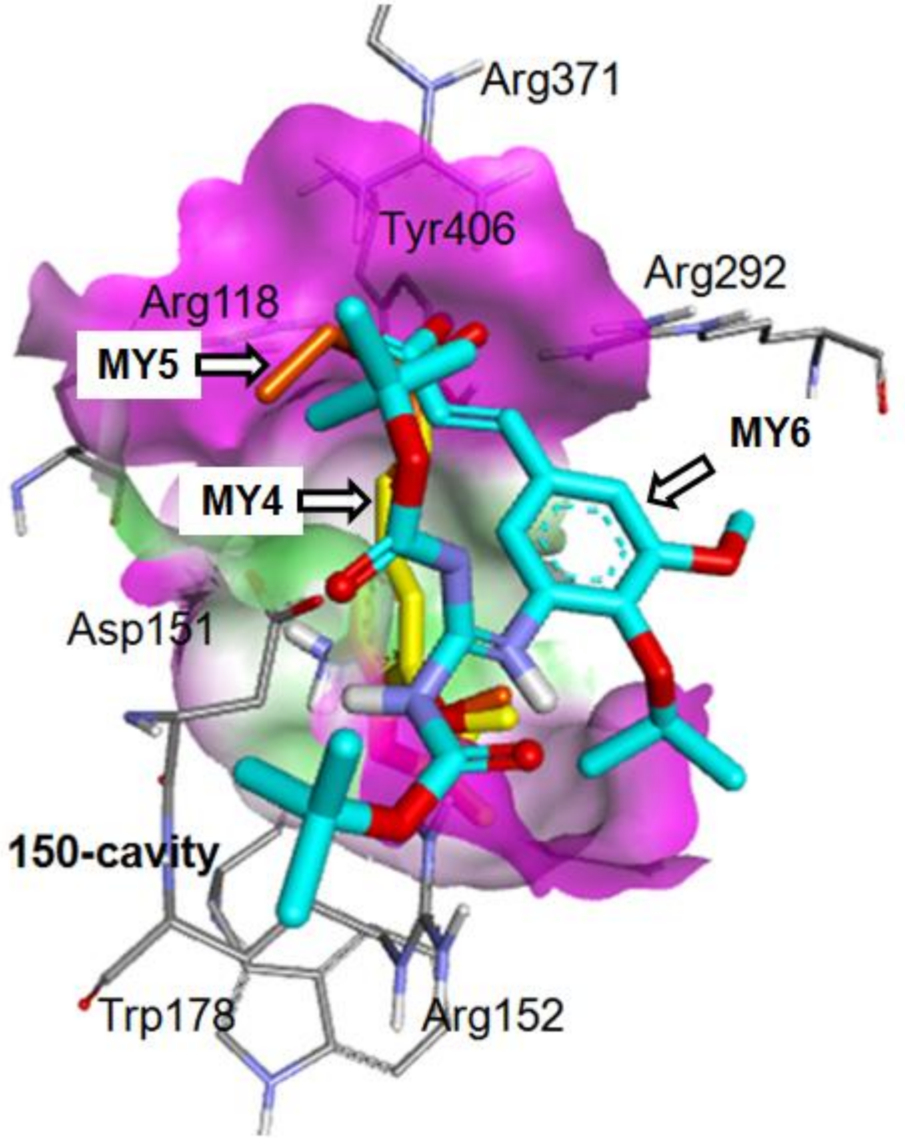

Figure S2. (a) The docked pose of **MY4**, **MY5 and MY6** onto NA's active site (carbons colored by yellow (**MY4**), orange (**MY5**), and light blue (**MY6**)

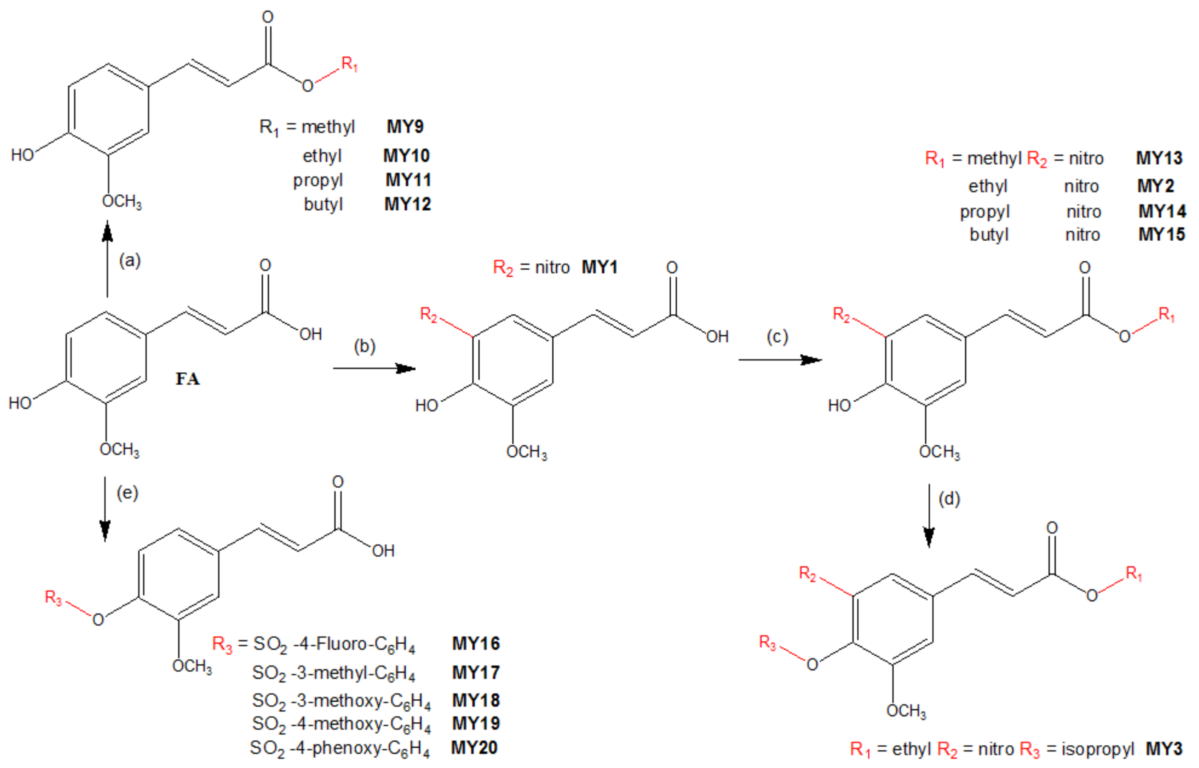

Figure S3. The synthesis scheme of ferulic acid derivatives. The reaction was conditioned as follows: (a) and (c) *n*-alcohol (methanol, ethanol, *n*-propanol, *n*-buthanol), H_2SO_4 98%, 80°C, 12 hours, (b) HNO_3 69%, glacial acetic acid, RT, 2 hours, (d) isopropyl bromide, TBAI, Cs_2CO_3, DMF, RT, 6 hours, (e) Ar-sulfonyl chloride pyridine, RT, 2 hours. The structure was drawn using ChemDraw Ultra 8.0 (www.chemdraw.com).

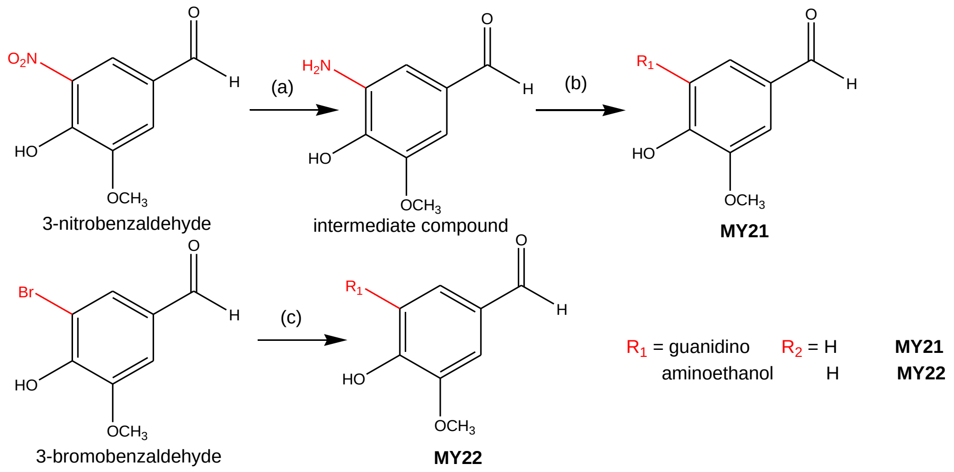

Figure S4. The synthesis scheme of vanillin derivatives. The reaction was conditioned as follows: (a) hydrazine hydrate, glyoxylic acid, methanol, RT, 1 hour, (b) cyanamide, HCl, 80°C, 6 hours, (c) 2-ethanolamine, DIPEA, DCM, RT, 24 hours. The structures were drawn using ChemDraw Ultra 8.0 (www.chembridgesoft.com).

concentration of 125 μg/mL

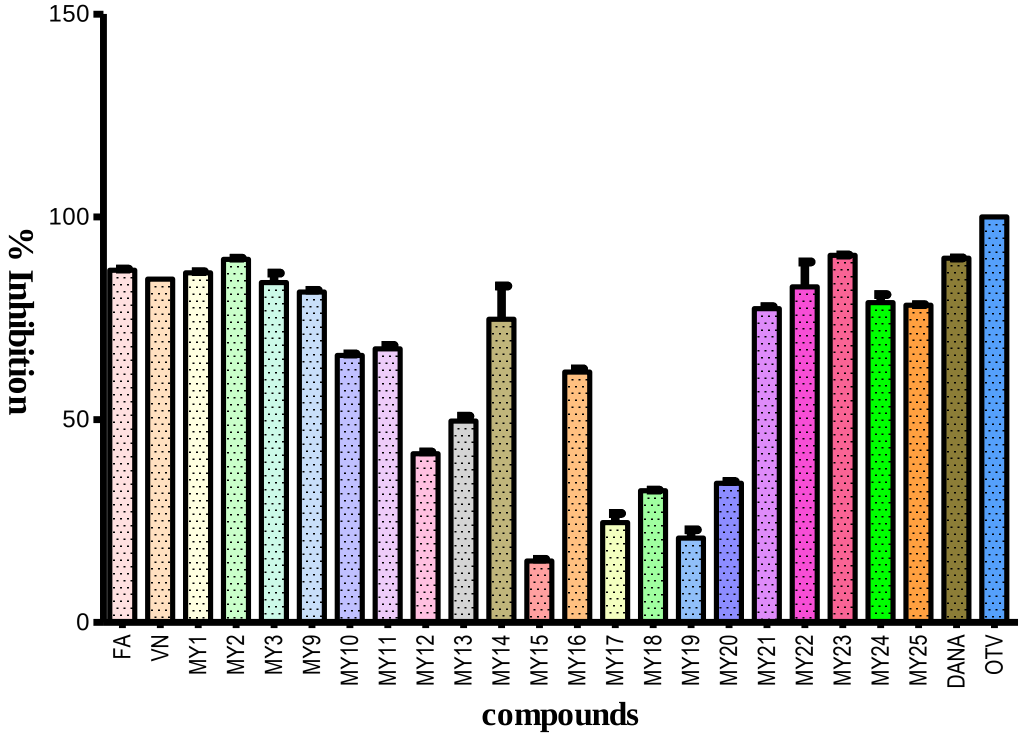

Figure S5. The percentage inhibition of **FA**, **VN** and **MY1-25** at 125 μg/mL. The graph was generated using GraphPad Prism 5.01 (www.graphpad.com).

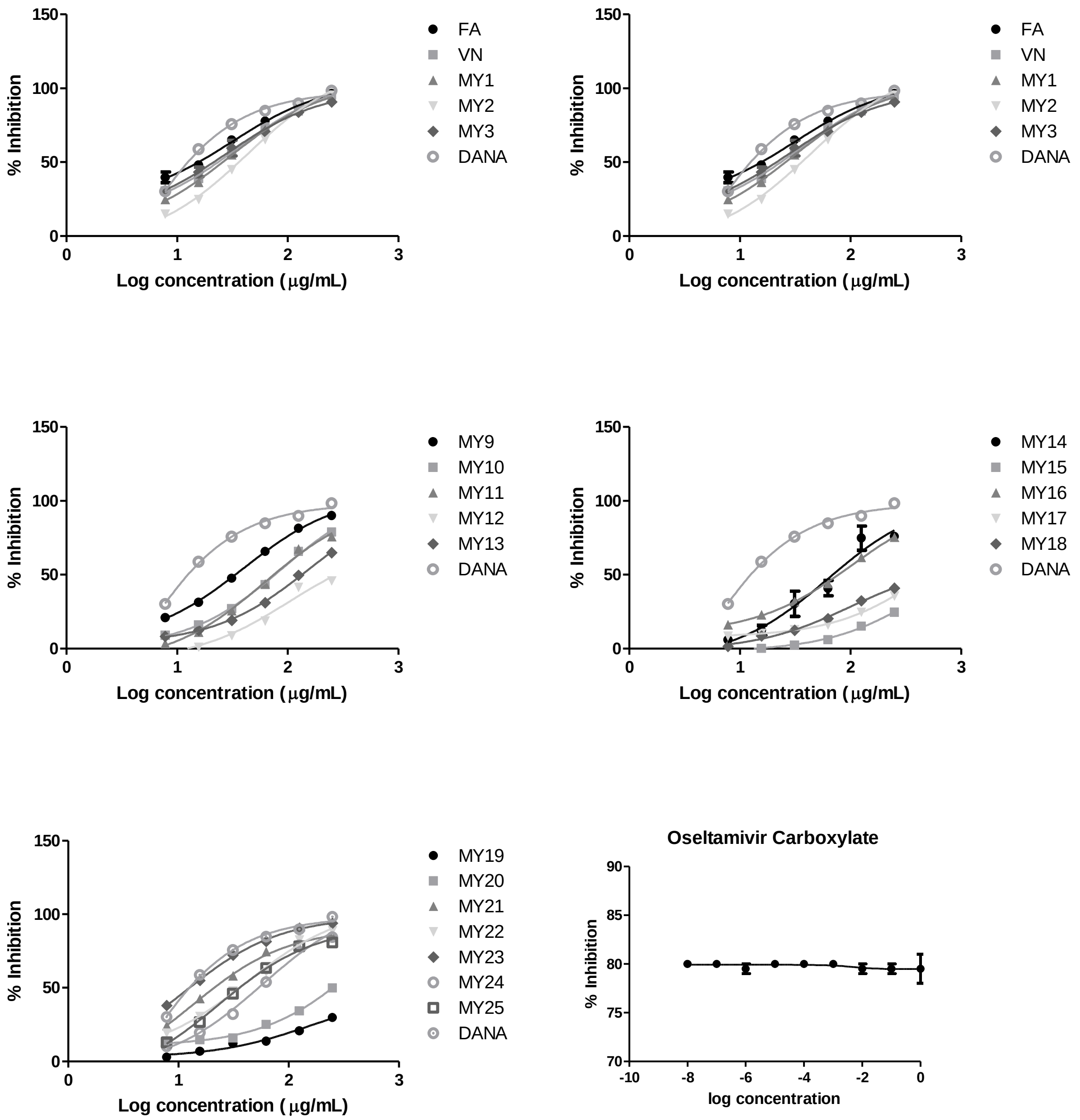

Figure S6.The percentage inhibition of H1N1NA of **FA**, **VN**, **MY1-3**, **M9-25**, **DANA and OTV**

References

1. Kiss, L. E., Ferreira, H. S., Torrao, L., Bonifacio, M. J., Palma, P. N., da-Silva, P. S., and Learmonth, D. A Discovery of a Long-Acting, Peripherally Selective Inhibitor of Catechol-*O*-methyltransferase. *J. Med. Chem.* **2010**, 53, 3405.

2. Moumne, R., Lavielle, S. and Karoyan, P, ., Efficient Synthesis of β2-Amino Acid by Homologation of α-Amino Acids Involving the Reformatsky Reaction and Mannich-Type Imminium Electrophile. *J. Org. Chem* **2006**, 71, 3332-3334.

3. Salvatore, R. N.; Smith, R. A.; Nischwitz, A. K.; Gavin, T., A Mild and Highly Convenient Chemoselective Alkylation of Thiols Using Cs_2CO_3–TBAI. *Tetrahedron Lett.* **2005**, 46, 8931-8935.
4. Marcotullio, M. C.; Campagna, V.; Sternativo, S.; Costantino, F.; Curini, M., A New, Simple Synthesis of *N*-tosyl Pyrrolidines and Piperidines. *Synthesis* **2006**, 2760-2766.
5. Raju, B., Ragul, R. and Sivasankar, B. N, A New Reagent for Selective Reduction of Nitro Group. *INDIAN J. CHEM* **2009**, 48B, 1315-1318.
6. Zhang, J.; Wang, Q.; Fang, H.; Xu, W.; Liu, A.; Du, G., Design, Synthesis, Inhibitory Activity, and SAR Studies of Hydrophobic *p*-Aminosalicylic Acid Derivatives as Neuraminidase Inhibitors. *Bioorg. Med. Chem.* **2008**, 16, 3839-3847.
7. Koley, M.; König, X.; Hilber, K.; Schnürch, M.; Stanetty, P.; Mihovilovic, M. D., Synthesis and Screening of 2, 6-Diamino-substituted Purine Derivatives as Potential Cardiomyogenesis Inducing Agents. *ARKIVOC* **2011**, 6, 45-61.
